# Examining elevated blood pressure and the effects of diabetes self-management education on blood pressure among a sample of Marshallese with type 2 diabetes in Arkansas

**DOI:** 10.1371/journal.pone.0250489

**Published:** 2021-04-22

**Authors:** Pearl A. McElfish, Christopher R. Long, Zoran Bursac, Aaron J. Scott, Harish E. Chatrathi, Ka‘imi A. Sinclair, Nirav Nagarsheth, Mikaila Calcagni, Jay Patolia, Marie-Rachelle Narcisse

**Affiliations:** 1 College of Medicine, University of Arkansas for Medical Sciences Northwest, Fayetteville, Arkansas, United States of America; 2 Department of Biostatistics, Robert Stempel College of Public Health and Social Work, Florida International University, Miami, Florida, United States of America; 3 Office of Community Health and Research, University of Arkansas for Medical Sciences Northwest, Fayetteville, Arkansas, United States of America; 4 College of Nursing, Washington State University, Seattle, Washington, United States of America; Emory University, UNITED STATES

## Abstract

**Introduction:**

Hypertension is a leading risk factor for heart attack and stroke. Undiagnosed hypertension increases the risk of heart attack and stroke. The risk of hypertension is increased for those with type 2 diabetes mellitus (T2DM). Diabetes self-management education (DSME) has been shown to be effective at improving clinical outcomes, including reducing blood pressure, but few studies have evaluated the effects of DSME for Native Hawaiians and Pacific Islanders.

**Methods:**

This study examined the baseline prevalence of diagnosed hypertension and undiagnosed high blood pressure and differences in health care access between those with diagnosed hypertension versus undiagnosed high blood pressure. The sample consisted of 221 Marshallese adults with T2DM participating in a DSME randomized controlled trial in northwest Arkansas. The study also examined the effects of DSME interventions on participants’ blood pressure, comparing an Adapted-Family DSME with a Standard DSME.

**Results:**

Nearly two-thirds of participants had blood pressure readings indicative of hypertension, and of those, over one-third were previously undiagnosed. The frequency of doctor visits was significantly lower for those with undiagnosed high blood pressure. There were no differences in health insurance coverage or forgone medical care between those with undiagnosed high blood pressure versus diagnosed hypertension. Across all participants, a significant reduction in systolic blood pressure occurred between baseline and post intervention, and a significant reduction in diastolic blood pressure occurred between baseline and post-intervention, 6 months, and 12 months post-intervention. No differences were observed by study arm.

**Conclusion:**

This study is the first to document the prevalence of diagnosed hypertension and undiagnosed high blood pressure, as well as the effects of DSME on blood pressure among a sample of Marshallese adults with T2DM.

## Introduction

Hypertension is one of the leading risk factors for heart attack and stroke. Over 100 million adults aged 20 years and older in the United States (US) have hypertension [1]. Minority populations, including blacks, Native Americans, and Native Hawaiians and Pacific Islanders (NHPI), have higher rates of hypertension compared with whites [2–4]. The risk of hypertension is increased for those who have type 2 diabetes mellitus (T2DM) [5,6].

More than 11 million adults in the US have hypertension that is undiagnosed [7]. Undiagnosed and untreated hypertension increases the risk of heart attack and stroke [8]. Undiagnosed hypertension is associated with age, education, income status, race/ethnicity, obesity, and health insurance status [9–11]. However, there has been limited research regarding its prevalence and associated risk factors among NHPI.

Despite the growing population of NHPI in the US, which has increased 40% from 2000 to 2010 [12], NHPI are underrepresented in health research, and many population-based studies do not report hypertension prevalence for NHPI. One exception is the 2014 NHPI National Health Interview Survey (NHPI NHIS), which found 29.8% of NHPI adults self-reported a previous hypertension diagnosis [4]. Analysis of state-level data in Hawaii demonstrated a prevalence of hypertension that was higher among NHPI than among Whites or Asians, documenting prevalence as high as 48% among NHPI [13–15].

Arkansas has experienced a significant increase in NHPI community members, most of whom are Marshallese. The growing Marshallese population, now estimated at more than 10,000 residents [16], is primarily located in northwest Arkansas. A study of Marshallese adults living in northwest Arkansas (*N* = 396) found 38.4% had T2DM and 41.2% had hypertension. Among those with blood pressure readings indicative of hypertension, 66.0% had not been previously diagnosed with hypertension by a healthcare professional [17].

Diabetes self-management education (DSME) has been shown to be effective at improving clinical outcomes, including reducing blood pressure [18,19]. However, few studies have examined the effects of DSME in NHPI communities [20,21]. The present study used data from a randomized controlled trial (RCT) comparing the effectiveness of a Standard DSME curriculum with an Adapted-Family DSME curriculum among Marshallese adults in northwest Arkansas. (See *Intervention Description* below.) Results of this RCT demonstrated significantly greater improvements in adjusted mean HbA_1c_ in the Adapted-Family DSME arm compared with the Standard DSME arm at post-intervention (Adj. Mean Diff. = -0.61%, *p* = 0.04) [22]. However, the effects of the DSME interventions on blood pressure have not been examined.

### Purpose of the study

The study aims to report the prevalence of diagnosed and undiagnosed hypertension among a sample of Marshallese adults with T2DM, explore if healthcare access is associated with lack of a previous diagnosis, and evaluate the effect of a culturally adapted DSME intervention on blood pressure.

## Methods

The RCT took place in Washington and Benton counties in northwest Arkansas. Bilingual Marshallese research staff recruited potential participants through community-driven methods which have been described elsewhere [22]. Participants were Marshallese adults (aged 18 and older) who had T2DM (defined as HbA_1c_ ≥6.5%). Eligible participants were provided information about the study in English and/or Marshallese and were provided opportunities to enroll in the study. Participants were consented and randomly assigned to one of two arms: the Adapted-Family DSME or the Standard DSME.

### Intervention description

The Standard DSME provided a total of 10 hours of education, covering eight core elements across sessions consistent with the American Diabetes Association (ADA) and American Association of Diabetes Educators’ (AADE) recommendations regarding self-care behaviors, including healthy eating, being active, managing stress, and taking prescribed medications [23]. Participants’ family members in the Standard DSME arm were not included in the education sessions. Sessions were conducted at a local non-profit center well known to participants and accessible to the Marshallese community.

The Adapted-Family DSME also provided 10 total hours and covered the same eight core elements of the Standard DSME. The Adapted-Family DSME was culturally adapted using a community-based participatory research (CBPR) approach to include culturally appropriate ontologies and examples. Participants were encouraged to invite family members to participate in the intervention, and the curriculum focused on the importance of engaging family members in behavioural changes. The Adapted-Family DSME was conducted in participants’ homes. The interventions were implemented between 2015 and 2018. A detailed description of each intervention has been published elsewhere [22,24].

Retention efforts across both study arms included bi-weekly participant email and phone contact. A $20 gift card was provided at each of the four data collection events (i.e., baseline, immediate post-intervention, 6 months post-intervention, and 12 months post-intervention.). The University of Arkansas for Medical Sciences Institutional Review Board approved the study (#203482), and it was registered at clinicaltrials.gov (#NCT02407132) and HSRProject (#HSRP20152031).

### Measurements

At each of the four data collection events, self-reported diagnosis of hypertension and health care access were obtained using items from the Behavioral Risk Factor Surveillance System (BRFFS) [25]. Self-reported diagnosis of hypertension was determined by the survey question “Has a doctor, nurse, or other health professional ever told you that you had hypertension?” Health care access was determined by the survey questions “Do you have any kind of health care coverage, including health insurance, prepaid plans such as HMOs, or government plans such as Medicare?” and “Was there a time in the past 12 months when you needed to see a doctor but could not because of cost?”

Trained study staff took participants’ blood pressure. Systolic blood pressure (SBP) and diastolic blood pressure (DBP) were measured with a sphygmomanometer and stethoscope or digital blood pressure device, with the participant seated and arm elevated. Participants were considered to have hypertension at baseline based on established diagnostic criteria: SBP ≥130 mm Hg or DBP ≥80 mm Hg [1].

### Sample

**Fig 1:**
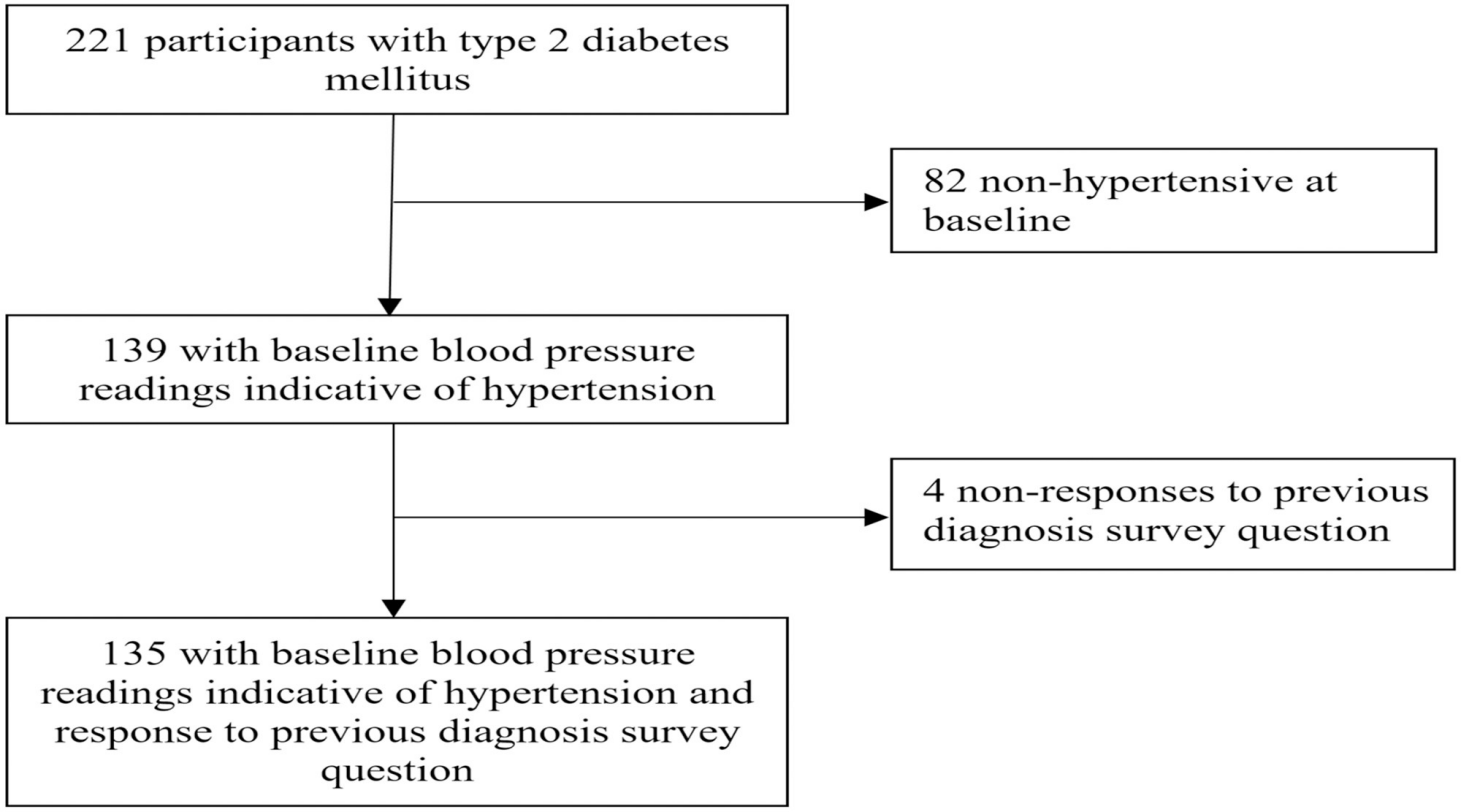
Participant sample flow diagram.

A total of 221 Marshallese adults with T2DM were enrolled. All of the enrolled participants were analysed to determine the prevalence of previously diagnosed hypertension and undiagnosed high blood pressure. Blood pressure readings were indicative of hypertension for 139 participants at baseline. All 139 participants with blood pressure readings indicative of hypertension at baseline were examined to determine if participation in DSME reduced blood pressure over time and/or differentially by intervention study arm. Of those 139 participants with blood pressure readings indicative of hypertension at baseline, 135 responded to the survey question regarding previous hypertension diagnosis. These 135 participants comprised the sample used to determine the prevalence of diagnosed hypertension and undiagnosed high blood pressure and to determine if there was an association between health care access and undiagnosed high blood pressure; See Fig 1 for the participant sample flow diagram.

### Statistical analysis

Descriptive statistics were used to determine the percentage of participants with hypertension and the percentage of participants with undiagnosed high blood pressure. The number of times patients visited a doctor, nurse, or other health professional in the past 12 months and hypertension diagnosis was examined using an independent t-test. Insurance coverage and foregone medical care due to cost and hypertension diagnosis were examined using chi-square tests. To determine if participation in DSME reduced blood pressure over time and/or differentially by intervention study arm, two linear mixed-effects regression models for repeated measures were fitted to estimate the impact on SBP and DBP. Three repeated time-point comparisons were used: baseline to 9-weeks, 6-months, and 12-months post-intervention.

Both regression models were adjusted for participant characteristics (sex, age, marital status, employment status, and education level). Study arm and control variables were treated as fixed effects, while time was treated as a random effect, with the assumption of unstructured covariance for both models. Interaction between study arm and time-points was tested within the model. All analyses were performed using SAS/STATv14.2 [26]. An a priori alpha of 0.05 was set as the two-tailed statistical significance level. Clinically meaningful improvements were defined as a reduction of 5 mmHg for SBP and 2 mmHg for DBP, which represent a 13% and 11.4% risk reduction of stroke, respectively, for individuals with T2DM [27]. Additionally, a 5 mmHg reduction in SBP has been found to reduce the risk of congestive heart failure (CHV) by 24% for those with T2DM [28].

## Results

Among the 221 participants enrolled in the DSME trial, 139 (62.9%) had blood pressure readings indicative of hypertension at baseline. Among those 139 participants, more than half (56.8%) were women, and the mean age was 54 years (SD = 10.5). Among the 135 with blood pressure readings indicative of hypertension and valid responses to the item regarding previous hypertension diagnosis, 52 (38.5%) had undiagnosed high blood pressure. Table 1 provides a summary of demographic characteristics and biometric measures.

**Table 1:** Descriptive statistics of demographics and biometric measures among participants with blood pressure readings indicative of hypertension.

Measure	Total (*n* = 139)	Family DSME (*n* = 74)	Standard DSME (*n* = 65)
Male	60 (43.2)	37 (50.0)	23 (35.4)
Employed	48 (34.5)	32 (43.2)	16 (24.6)
Married	99 (71.2)	52 (70.3)	47 (72.3)
Education			
Less than HS Diploma/GED	83 (59.7)	48 (64.9)	35 (53.9)
HS Diploma/GED	39 (28.1)	19 (25.7)	20 (30.8)
More than HS Diploma/GED	17 (12.2)	7 (9.5)	10 (15.4)
Age†	54±10.5	52.5±10.0	56.1±10.8
SBP†			
Baseline	145.6±19.1	143.4±18.9	148.0±19.3
9 Weeks	139.2±22.3	137.6±23.2	141.0±21.4
6 months	140.86±24.9	141.9±25.6	139.6±23.9
12 months	138.3±23.0	135.1±21.9	141.6±23.9
DBP†			
Baseline	83.9±10.8	84.1±10.6	83.8±11.2
9 Weeks	79.4±10.5	79.5±9.9	79.3±11.2
6 months	78.3±10.9	79.8±10.5	76.4±11.2
12 months	77.7±11.2	76.9±10.7	78.5±11.7

Values are *n* (%) unless otherwise indicated;

^†^Mean±Standard Deviation; DSME = diabetes self-management education; HS = high school; GED = general education diploma; SBP = systolic blood pressure; DBP = diastolic blood pressure.

Participants with undiagnosed high blood pressure reported seeing the doctor significantly fewer times per year (*Mean* = 1.96, *SD* = 2.60) compared with those with previously diagnosed hypertension (*Mean* = 3.37, *SD* = 3.38) (*t = 2*.*73*, *p*<0.01). There were no statistically significant differences based on insurance coverage or forgone medical care between those with undiagnosed high blood pressure versus those with previously diagnosed hypertension.

In the first two mixed-effects models, the interaction between study arm and time was not significant for SBP (*p* = 0.26) or DBP (*p* = 0.06). Therefore, it was not included in subsequent models. Parameter estimates resulting from the two final mixed-effects regression models are provided in Table 2.

**Table 2:** Mixed effects regression models: Systolic and diastolic blood pressure.

Measure	*B*	*SE*	*df*	*t*	*p*
*Systolic Blood Pressure*					
Study arm	2.69	2.64	132	1.02	0.31
Baseline^†^	-	-	-	-	-
9 weeks^††^	-5.83	1.94	132	3.00	**0.02**
6 months^††^	-3.69	1.92	132	1.95	0.34
12 months^††^	-5.29	2.07	132	2.55	0.07
*Diastolic Blood Pressure*					
Study arm	0.52	1.45	132	0.36	0.72
Baseline^†^	-	-	-	-	-
9 weeks^††^	-4.38	1.07	132	4.11	**<0.001**
6 months^††^	-5.56	1.02	132	5.47	**<0.001**
12 months^††^	-6.17	1.11	132	5.57	**<0.001**

Note: Each model includes age, sex, marital status, employment status, and education level as control variables.

^†^Baseline is the reference category;

^††^Bonferroni adjusted values for 9 week, 6 month, and 12 month post intervention.

There was no significant study arm by time interaction in either model. Marginal time effects are reported.

First, among all participants with blood pressure readings indicative of hypertension at baseline (*n* = 139), there was a significant decrease in systolic blood pressure between baseline and immediate post-intervention (*B* = -5.83, *p* = 0.02), but not between baseline and 6-month (*p* = 0.34) or 12-month post-intervention (*p* = 0.07). However, there was no statistically significant difference in systolic blood pressure between study arms (*p* = 0.31). Second, a significant decrease in diastolic blood pressure between baseline and immediate post-intervention (*B* = -4.38, *p*<0.001), 6-month (*B* = -5.56, *p*<0.001), and 12-month (*B* = -6.17, *p*<0.001) post-intervention was achieved; however, there was no statistically significant difference between study arms (*p* = 0.72).

## Discussion

Marshallese with T2DM had a high prevalence of diagnosed hypertension or undiagnosed high blood pressure (62.9%), more than three times the prevalence of hypertension (19.2%) recorded in the only prior study reporting hypertension (≥140/90) among Marshallese with T2DM [17]. Among those with blood pressure readings indicative of hypertension, prevalence of undiagnosed high blood pressure was found to be 38.5% among Marshallese with T2DM, which is higher than the undiagnosed hypertension (≥140/90) documented in other populations [9,29–32].

This study found that participants with T2DM and undiagnosed high blood pressure visited the doctor significantly less frequently than those who had been previously diagnosed with hypertension. However, this study did not find significant differences between having diagnosed hypertension or undiagnosed high blood pressure and insurance status. This finding is in contrast to prior research from the National Health and Nutrition Examination Survey (NHANES) that showed 30% of hypertensive adults without health insurance were unaware of their hypertension compared with 14.4% of those with health insurance [11]. This study’s findings suggest barriers beyond insurance status are constraining hypertension diagnosis in the present sample. Prior literature suggests that language barriers may reduce appropriate diagnosis in other populations [33], and several studies have demonstrated language barriers among Marshallese receiving care in the US [34–36]. Additional research is needed to understand if language barriers are associated with undiagnosed high blood pressure.

The high prevalence of undiagnosed high blood pressure paired with reduced frequency of health care provider visits for those with undiagnosed high blood pressure poses a significant concern, because hypertension is a leading risk factor for heart attack and stroke [1,37], and prior literature has demonstrated that NHPI have an increased rate of stroke morbidity and mortality [14,38,39]. Furthermore, prior literature among NHPI has documented limited knowledge related to heart attack and stroke [40]. The high risk related to undiagnosed high blood pressure combined with a lack of heart attack and stroke knowledge demonstrates a need for accessible hypertension screening and culturally appropriate health education among Marshallese.

Consistent with prior literature in other populations, exposure to DSME improved blood pressure [41]. While this improvement was observed for SBP at statistically significant and clinically meaningful levels at post-intervention only, improvements in DBP more than doubled clinically meaningful levels and persisted over time. This study makes a significant contribution to the literature as the first study to demonstrate the effect of DSME on blood pressure in a sample of Marshallese adults with T2DM. Many of the components of the DSME curriculum align with suggested lifestyle modifications for reducing blood pressure, including reducing weight, healthy eating, being active, and managing stress [42].

### Limitations & strengths

This study only included Marshallese participants with T2DM living in Arkansas and cannot be generalized to Marshallese living outside of Arkansas, other populations, or those without T2DM. The study sample was limited to those who had blood pressure readings indicative of hypertension at baseline. This led to a slight underestimate of hypertension prevalence in the overall sample, as any participants with a previous diagnosis of hypertension but currently controlled blood pressures were excluded from analyses. Furthermore, the analytic sample was refined based on self-reported previous diagnosis of hypertension. Although self-reported hypertension status is not as accurate as medical records and/or laboratory measures, it is a commonly used measure in population-based studies such as the BRFSS, NHANES, and NHIS and has been validated in numerous races/ethnicities as well as in other countries [43–49]. Additionally, the original study design was not powered to test hypotheses regarding undiagnosed high blood pressure but was powered to detect changes in HbA_1c_. Similarly, because the primary outcome of the study was change in HbA_1c_, information regarding current blood pressure medication was not captured and therefore could not be controlled for in the current analyses. Finally, the intervention was aimed at improving the management of T2DM, rather than high blood pressure. Future studies should be powered to test for differences in blood pressure outcomes between various treatments, each designed specifically to reduce blood pressure through a variety of educational and health care options.

Despite these limitations, this is the first study to document diagnosed hypertension and undiagnosed high blood pressure among Marshallese with T2DM and the effect of DSME on blood pressure. This study makes a significant contribution to the literature on an understudied population facing stark health disparities.

## Supporting information

S1 Dataset Truncated dataset underlying the results. (XLSX)
